# Mpox in children and adolescents and contact follow-up in school settings in greater Paris, France, May 2022 to July 2023

**DOI:** 10.2807/1560-7917.ES.2024.29.21.2300555

**Published:** 2024-05-23

**Authors:** Laura Reques, Lilas Mercuriali, Yassoungo Silué, Emilie Chazelle, Guillaume Spaccaferri, Annie Velter, Alexandra Mailles, Pierre Frange, Arnaud Tarantola

**Affiliations:** 1 Health Surveillance and Safety Department, Regional Health Agency in the Île-de-France Region (Agence Régionale de Santé d’Île-de-France), Saint-Denis, France; 2 Santé publique France regional office, Saint-Denis, France; 3 Santé publique France, the national public health agency, Saint-Maurice, France; 4 Infection Control Unit, Laboratory of Clinical Microbiology, Necker - Enfants malades Hospital, AP-HP, Université Paris Cité, Paris, France

**Keywords:** Mpox, surveillance, paediatric, contact tracing, vaccination

## Abstract

**Background:**

During the 2022 mpox outbreak in Europe, primarily affecting men who have sex with men, a limited number of cases among children and adolescents were identified. Paediatric cases from outbreaks in endemic countries have been associated with a higher likelihood of severe illness. Detailed clinical case descriptions and interventions in school settings before 2022 are limited.

**Aim:**

To describe clinical characteristics of mpox cases among children (< 15 years) and adolescents (15–17 years) in the greater Paris area in France, and infection control measures in schools.

**Methods:**

We describe all notified laboratory-confirmed and non-laboratory-confirmed cases among children and adolescents identified from May 2022 to July 2023, including demographic and clinical characterisation and infection control measures in school settings, i.e. contact tracing, contact vaccination, secondary attack rate and post-exposure vaccination uptake.

**Results:**

Nineteen cases were notified (13 children, 6 adolescents). Four adolescent cases reported sexual contact before symptom onset. Ten child cases were secondary cases of adult patients; three cases were cryptic, with vesicles on hands, arms and/or legs and one case additionally presented with genitoanal lesions. Five cases attended school during their infectious period, with 160 at-risk contacts identified, and one secondary case. Five at-risk contacts were vaccinated following exposure.

**Conclusion:**

Cases among children and adolescents are infrequent but require a careful approach to identify the source of infection and ensure infection control measures. We advocate a ‘contact warning’ strategy vs ‘contact tracing’ in order to prevent alarm and stigma. Low post-exposure vaccination rates are expected.

**Key public health message**

**What did you want to address in this study and why?**

Before 2022, mpox, a disease similar to smallpox causing fever and a skin rash, mainly occurred in Africa and was associated with severe manifestations in children. The 2022–23 outbreak in Europe mostly affected men who had sex with men. Cases among children and adolescents were rare. We aimed to describe these mpox paediatric cases in greater Paris, France, as well as outbreak control measures in school settings to inform future public health measures.

**What have we learnt from this study?**

Nineteen cases were notified during the study period and 160 at-risk contacts were investigated and followed-up in school settings. Mpox cases among children were mainly linked to an infected person within the household, while adolescents reported sexual contact before they developed symptoms. Transmission events between children within school settings were very infrequent and post-exposure vaccination uptake was very low.

**What are the implications of your findings for public health?**

Children and adolescents who showed mpox symptoms were rare. We recommend a careful approach to identify the source of infection and to disseminate the adequate infection control messages in school settings. Because of the stigma associated with the disease, a tailored communication strategy to inform the school community without alarm is advised.

## Introduction

Monkeypox virus (MPXV) is an *Orthopoxvirus* responsible for mpox, a disease endemic in West and Central Africa. Although historically a zoonosis, MPXV Clade 2b was responsible for a global outbreak of sexually transmitted mpox affecting mainly men who have sex with men (MSM) in America and Europe in spring and summer 2022 [1].

Upon detection of the first locally acquired cases in the United Kingdom (UK) and Spain, on 17 May 2022 the French surveillance system moved from routine mandatory notification of orthopoxvirus-infected cases to an enhanced surveillance of mpox [2]. The objectives of this surveillance were to detect and isolate cases as well as to identify, trace and monitor their contacts. Cases were investigated and invited to name their at-risk contacts for contact tracing, who were offered post-exposure vaccination with third-generation smallpox vaccine (Modified Vaccinia Ankara – Bavarian Nordic (MVA-BN) from 25 May 2022 onwards [3]. Recommendation of post-exposure vaccination was extended to children from 20 June 2022 [4].

In France, most cases were notified between 17 May and 30 September 2022, with a peak of cases by onset date on 1 July. Most common symptoms included rash (95%), mainly in the genital and perianal area, and fever > 38° C (71%). The proportion of severe cases was very low (hospitalisation was documented for 3% of cases) and no deaths were documented. Cases were mostly in men (97%), were relatively young (mean age: 37 years) and transmission was primarily through close physical contact or sexual networks of MSM. Cases among heterosexual men, women and children were sporadic [5]. 

The Île-de-France region (IdF), which includes Paris and greater Paris area and has a population of 12.3 million habitants, was the most affected region of France during this outbreak. Between May 2022 and July 2023, 3,112 mpox cases (2,502 laboratory-confirmed and 610 non-laboratory-confirmed) were notified in IdF, representing 63%, 61% and 72% of the total, the laboratory-confirmed and the non-laboratory-confirmed cases documented in France, respectively [6].Detailed descriptions of mpox infections in children and adolescents were limited before the 2022 outbreak. Since the first identified human case in a 9-month-old boy in 1970 [7], mpox has been associated with children rather than adults in endemic areas where Clade 1 and Clade 2 circulate [8,9], and paediatric cases had a higher likelihood of severe illness and mortality [10]. However, a significant proportion of cases in endemic areas may be of zoonotic origin, and local household characteristics, i.e. lack of hygiene and crowding, facilitate transmission. Recent studies have shown that the proportion of children and adolescents was significantly lower in the 2022–23 mpox outbreak [11,12] and that severe cases were rare [13]. We aimed to describe the characteristics of all mpox cases among children (< 15 years) and adolescents (aged 15–17 years) in the IdF region, secondary transmission in school settings and post-exposure vaccination uptake in order to inform future public health measures. 

## Methods

### Study setting and population

In France, diseases caused by *Orthopoxviruses* including mpox are submitted to mandatory notification. Health clinicians and laboratories report all mpox cases to regional health agencies (RHA). Staff from RHA and the national public health agency Santé publique France interview cases using a standardised questionnaire [6].

Our study is based on data concerning all reported mpox cases under 18 years of age, considering children as cases under 15 years old and adolescent cases as aged between 15 and 17 years, inclusive. The study period included all cases notified between May 2022 and July 2023 in IdF.

### Case and contact definitions

According to the national case definition [6], laboratory-confirmed mpox infection included an MPXV-positive real-time PCR test or a positive generic orthopoxvirus real-time PCR result. Non-laboratory-confirmed cases included an individual presenting with a rash suggestive of mpox on any part of the body (including genital/perianal, oral) who also: (i) had an epidemiological link to a confirmed mpox case in the 3 weeks before symptom onset (probable case) or (ii) is MSM, or any person regardless of gender or sexual orientation who, in the 3 weeks before symptom onset, had two or more sexual partners, or had travelled to an endemic country (possible case). The rash could be either isolated, i.e. with no other symptoms, or preceded by or accompanied with fever (> 38 °C), swollen lymph nodes (lymphadenopathy) or pain when swallowing (odynophagia). From 8 July 2022, laboratory confirmation of probable cases and possible cases (after exclusion of differential diagnosis) was not necessary because of the case definition’s high positive predictive value, but notification to RHA remained compulsory.

At-risk contacts were defined as individuals who had direct, unprotected contact with the compromised skin or fluids of a laboratory-confirmed or non-laboratory-confirmed symptomatic case, including through sharing personal items. Additionally, prolonged unprotected contact within 2 m for at least 3 h with a symptomatic case was also considered a potential risk. 

### Data collection

Physicians collected lesion swabs from suspected mpox cases. Initially, biological case confirmation was done by the national reference laboratory (in Brétigny sur Orge), but subsequently also by hospital laboratories, and private laboratories nationwide [14].

Collected information included region of notification, age and sex, human immunodeficiency virus (HIV) status, HIV pre-exposure prophylaxis (PrEP) use, clinical characteristics, sexual orientation and behaviour, and specific exposures in the 3 weeks before symptom onset including travel history [6]. The questionnaires were filled out by adolescents and by children’s parents or legal guardians. 

### Recommendations for cases and at-risk contacts

French guidelines for case management recommended isolation for a period of 3 weeks after symptom onset, avoiding public gatherings, wearing a surgical mask, covering skin lesions and abstaining from sexual intercourse. Condom-wearing during sexual intercourse was advised for up to 8 weeks after the end of isolation [6]. 

Official recommendations for at-risk contacts included self-monitoring up to 21 days and vaccination with the third-generation modified vaccinia virus Ankara vaccine up to 14 days after the last exposure to a case (ideally within 4 days) [3]. 

On 20 June 2022, French authorities extended post-exposure vaccination recommendations to children under the age of 18, but only after a careful risk-benefit assessment given the absence of clinical data on the safety of the third-generation vaccines in this age group. This assessment had to be carried out by medical specialists, within the framework of a shared medical decision and with the consent of the minor’s legal guardian [4].

### Investigations in school settings

Investigations in school settings were coordinated by the RHA, with the cooperation of health school teams (school doctor, nurses and psychologists) and local authorities (town hall services, medical centres in proximity and reference hospitals).

As per national definitions and guidelines, mpox cases attending all school settings (daycare to high school) triggered contact tracing and infection control interventions, including frequent handwashing, appropriate disinfection of contaminated surfaces and improved ventilation. School closure was not recommended.

School exclusion for 3 weeks from symptom onset was recommended for all cases. At-risk contacts were identified as all classmates, teachers and children who shared personal items, transportation, eating spaces (canteen, refectories) or extracurricular activities with an index case implying direct contact. Identified at-risk contacts were offered specialised medical advice, testing in case of symptoms and vaccination, if eligible; school exclusion was not recommended to at-risk contacts. 

For each school, a nearby vaccination centre was identified and at-risk contacts had a priority referral. At-risk contacts were followed-up for 21 days by RHA and school health teams (weekly contact by telephone to the family).

### Statistical analysis

Descriptive analysis included demographic and clinical characterisation of cases, contact tracing and vaccination, and secondary attack rate with 95% Poisson confidence interval. Data on index cases, secondary cases and contacts were entered using Excel (Microsoft Corp.) and analysed using Stata 13 (Stata Corp.). 

## Results

**Figure 1:**
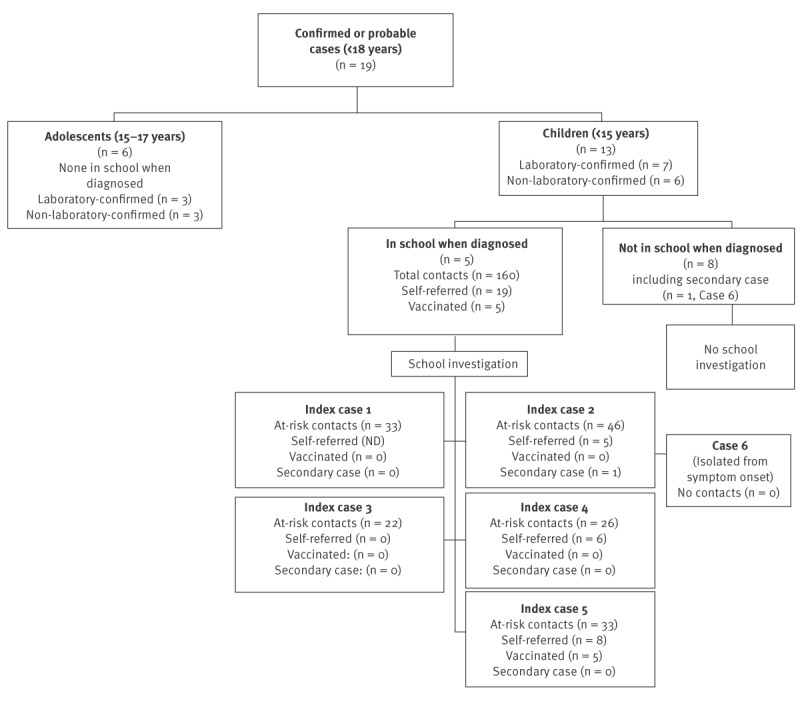
Data flowchart of laboratory-confirmed and non-laboratory-confirmed mpox cases aged below 18 years and investigations in schools, greater Paris area, France, May 2022–July 2023 (n = 19)

**Figure 2:**
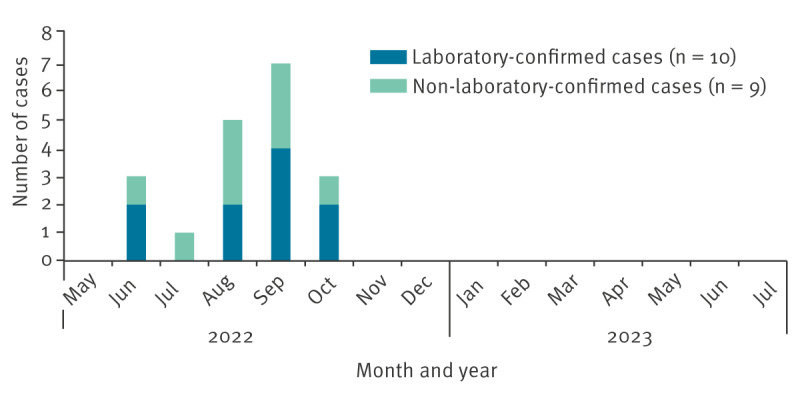
Epidemiological curve of laboratory-confirmed and non-laboratory-confirmed mpox cases aged below 18 years, greater Paris area, France, May 2022–July 2023 (n = 19)

Between May 2022 and July 2023, 19 cases aged below 18 years were reported in IdF (10 laboratory-confirmed and 9 non-laboratory-confirmed) (Figure 1), representing 50% of national cases in children and adolescents in this period (n = 38) [2]. Four cases were reported before 31 July 2022, 12 cases between 1 August and 30 September 2022 and three cases after 1 October 2022 (Figure 2).

ND: not determined.

Self-referred at-risk contacts were given the name of a referral centre and they and their families had priority in accessing medical advice and vaccination, but they alone decided whether or not to seek care. Vaccination was offered with third-generation smallpox vaccine (Modified Vaccinia Ankara – Bavarian Nordic (MVA-BN)).

### Case characteristics

The median age of the cases was 5.5 years (interquartile range (IQR): 3.2–17.0). All cases were symptomatic at the time of diagnosis. The median delay between the onset of symptoms and testing was 3.5 days (IQR: 3.0–10.0). For all cases, contact tracing and case management measures were initiated on the same day or the day after notification to RHA, with the exception of one case, who was contacted on the third day after notification. All specimens were obtained from cutaneous lesions. None of the patients received antiviral treatment. One case required hospital care for a skin lesion bacterial superinfection, but all cases had a favourable outcome. None of the cases had previously been immunised against smallpox.

Six cases were identified among adolescents aged 15 to 17 years. Three were laboratory-confirmed and three were non-laboratory-confirmed, one of which was epidemiologically linked to a confirmed adult case. Three cases were male (none of them identified as MSM) and three cases were female. The median age was 17.1 years (IQR: 17.0 – 17.6). Two cases presented fever before lesion onset. Sexual contact within the 3 weeks preceding the symptom onset was documented for four cases. Genital and/or anal lesions were present in five cases, suggesting a sexually transmitted infection. In one case, the location of the lesions was undocumented.

Thirteen cases were documented among children aged 0 to 14 years. Seven were laboratory-confirmed and six were non-laboratory-confirmed, all of whom were epidemiologically linked to a confirmed case. Eight cases were male, and five cases were female. The median age was 3.7 years (IQR: 2.4–5.5). Six cases presented fever before lesion onset. Three were cryptic cases i.e. no identified origin of infection, with vesicles on hands, arms and/or legs. One of the three additionally presented with isolated lesions of the genitoanal area, which resulted in an alert to social services for investigation. The remaining 10 cases – including three pairs of siblings – were all secondary cases of known adult cases and likely contracted the virus within the household. Four cases presented lesions on arms, four cases on legs and one case on the face. Two cases presented lesions on the buttocks but no genital or anal lesions.

### Investigation of at-risk contacts in school settings

Of the 19 mpox cases, five had attended school during their infectious period and were investigated (median age: 5.5 years; IQR: 3.7 – 6.5; range: 3.5 – 7.0). The cases attended five different schools in four departments of the greater Paris area. Two investigations took place in June, two in September and one in October. Schools were closed from 7 July to 5 September for the summer holidays. 

The five index cases had a total of 160 at-risk contacts (Figure 1). Among these, 113 were classmates, 23 were school professionals and 24 shared other activities (transportation, canteen or extracurricular activities). Medical advice by a paediatric infectious disease specialist was proposed to all. Among the 128 at-risk contacts potentially eligible for post-exposure vaccination, 19 (12%) were self-referred to the specialists and five (4%) accepted vaccination. One at-risk contact developed symptoms during the 3-week follow-up and tested positive, giving a secondary attack rate of 0.65% (Poisson 95% CI: 0.09–4.44). This at-risk contact (Case 6 in Figure 1) was not a classmate but had had direct contact with the index case through an extracurricular activity and had not been vaccinated following exposure. During the case investigation, no other possible source of infection was found.

## Discussion

Children and adolescents have accounted for 0.6% of cases in greater Paris during the 2022 mpox outbreak, close to the rate (0.3%) described in the United States (US) and in Europe during the same period [15,16]. This relatively low proportion of MPXV infections among children and adolescents in 2022 is likely attributable to Clade IIb being predominantly transmitted through human-to-human intimate physical contact in this outbreak. However, because asymptomatic infections have been described in adults with percentages estimated between 6.5 and 14% [17,18] and global surveillance data suggest that the severity of Clade IIb infection in children is low [19], we cannot rule out that the incidence of mpox disease was underestimated among children during the 2022 outbreak.

Most of the reported mpox cases in adolescents presented a clinical picture, evolution and risk factors (sexual behaviour) similar to those of adults, with the exception that boys did not self-declare as MSM, which is consistent with literature [15,20]. The other cases were children aged below 15 years, one of whom was a secondary case in the school setting. The secondary attack rate in five school investigations was very low at 0.65%, although paucisymptomatic cases may have not been identified if families or caregivers decided against seeking medical advice and testing. Mpox was therefore not found to be highly contagious among children and adolescents in the French context. The risk of transmission linked with contacts within households has been estimated at 7.2% among unvaccinated individuals in Democratic Republic of the Congo [21] and at 1.9% (IQR: 0.4–5.6) in France [22]. Similarly, children who were attending school when diagnosed did not overwhelmingly transmit the infection to others. A national study from the UK undertook school investigations around four paediatric index cases, with 340 students and 100 staff presumably exposed, and found no secondary cases, giving an estimated secondary attack rate with a Poisson interval ranging 0–0.02% among children [23]. Another study conducted in the US including 10 paediatric cases who attended school did not find any secondary case, although no denominators were provided [15].

Concerning possible sources of infection and clinical presentation, for several of the 19 investigated cases, the source of infection was unknown, and one case presented genitoanal lesions. Public health and childhood protection workers must exercise due diligence when handling such cases. Nevertheless, it is important to consider that false positive MPXV test results are more frequent in low-prevalence populations. Furthermore, MPXV may have tropism for mucosa, regardless of the mode of inoculation, including diapering [15]. In the prospective cohort study conducted in Spain, a low but notable percentage of MSM presented anal lesions despite not engaging in anal-receptive sex [24]. Individuals with atypical presentations but also people who are completely asymptomatic might have a role in spreading and sustaining MPXV circulation [25]. Thus, silent transmission within the household must not be excluded.

In our context, the number of contacts who sought medical advice and vaccination after a case of mpox in a school setting was low (12% and 4%, respectively). Even if we had chosen an exhaustive contact tracing and follow-up strategy (individual contact, weekly follow up, specialised medical advice and vaccination proposal), the outcome may not have been different in terms of medical management and post-exposure vaccination. Other similar studies have also found low post-exposure vaccination rates in school settings (11%) [26]. This fact can be linked to minimisation of risk of infection/lack of awareness by parents, fear of stigmatisation or secondary effects of the vaccine, distance to regional surveillance teams who ensured the contact tracing and follow-up, or the decision by some families to seek medical advice elsewhere. In addition, given that mpox was considered a rare disease and mainly linked to young men during the current outbreak, our strategy could create alarm within the school community. These facts highlight that a ‘contact warning’ approach would be more suitable in order to prevent alarm and stigmatisation, including the engagement of school medical teams and teachers to spread an adapted message to families and a more active role of these actors in the follow-up and orientation for vaccination. Complementary actions should be considered in order to improve compliance with infection control measures in the event of a new epidemic, especially if it involved a more transmissible or more pathogenic virus.

Our study may have several limitations. Firstly, there is a risk of underdiagnosis and undernotification as the paediatric population was not the most affected during this outbreak and was therefore not targeted by information and prevention campaigns. Undernotification in the general population is suspected because of mpox detection in wastewater in France and Spain, although a small number of cases had been reported [27]. Secondly, cases may have been underdiagnosed on account of mild symptoms or asymptomatic infections. Available data, however, point to few asymptomatic infections among diagnosed mpox cases [28]. Some cases may have been wrongly attributed to other diseases, such as chickenpox, given the concurrent seasonal chickenpox epidemic, which peaked between 16 and 22 May 2022 [29]. Thirdly, the secondary attack rate may have been overestimated if the identified secondary case had acquired the infection elsewhere. However, this is unlikely, since their symptoms appeared within the incubation period of the last contact to the case, the fact that they had been advised to reduce social interactions during that period and the absence of other known exposures. Fourthly, all schoolmates and peers of cases were considered a priori contacts, with no contact gradation. Closeness, however, is difficult to estimate retrospectively, especially among young children. Fifthly, in the absence of molecular typing, the possibility that the secondary case acquired the disease in another setting cannot be formally excluded, although contact tracing around the secondary case did not show an alternative explanation and MPXV infections remained very rare in the general population. Additionally, most cases were identified during the summer school break, which resulted in a small number of cases being in a collective setting during their infectious period. Finally, it would be suitable to explore reasons for vaccination refusal. 

## Conclusion

During the mpox outbreak in 2022 in France, symptomatic paediatric cases were infrequent in greater Paris. A careful approach was required to identify the source of infection, as well as to orient and follow-up school at-risk contacts. The risk of infection among children is mainly linked to close intrafamilial contact. Our and other data on transmission through casual contact, however, justify a reasonable and evidence-based strategy in schools of limiting intervention to simply informing exposed children’s parents and steering them towards specialised care and prevention if they wish. We would recommend a ‘contact warning’ strategy over ‘contact tracing’ with an expectably low vaccine uptake.
